# Developmental constraints on fin diversity

**DOI:** 10.1111/dgd.12670

**Published:** 2020-06-01

**Authors:** Alyssa Enny, Kathleen Flaherty, Shunsuke Mori, Natalie Turner, Tetsuya Nakamura

**Affiliations:** ^1^ Department of Genetics Rutgers the State University of New Jersey Piscataway NJ USA; ^2^ Rutgers Animal Care Rutgers the State University of New Jersey Piscataway NJ USA

**Keywords:** developmental constraints, diversity, evolution, fin, skeleton

## Abstract

The fish fin is a breathtaking repository full of evolutionary diversity, novelty, and convergence. Over 500 million years, the adaptation to novel habitats has provided landscapes of fin diversity. Although comparative anatomy of evolutionarily divergent patterns over centuries has highlighted the fundamental architectures and evolutionary trends of fins, including convergent evolution, the developmental constraints on fin evolution, which bias the evolutionary trajectories of fin morphology, largely remain elusive. Here, we review the evolutionary history, developmental mechanisms, and evolutionary underpinnings of paired fins, illuminating possible developmental constraints on fin evolution. Our compilation of anatomical and genetic knowledge of fin development sheds light on the canalized and the unpredictable aspects of fin shape in evolution. Leveraged by an arsenal of genomic and genetic tools within the working arena of spectacular fin diversity, evolutionary developmental biology embarks on the establishment of conceptual framework for developmental constraints, previously enigmatic properties of evolution.

## Graphical abstract

The fish fin is a breathtaking repository full of evolutionary diversity, novelty, and convergence. However, the genetic underpinnings of their diversity and convergent evolution largely remain elusive. We summarized recent discoveries of evolutionary and developmental mechanisms of fins, shedding light on developmental constraints for fin diversity.

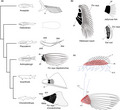

## 1 INTRODUCTION

Morphological diversity is a central facet enabling species to occupy new habitats. Fish with paired fins are ecologically and evolutionarily the most diversified group in vertebrates. They exhibit a full spectrum of morphological diversity which allows them to inhabit diverse environments from 8,000 m deep up to the surface of the ocean, providing ambulatory locomotion and even flight above the water in some species (Nelson, 2006). A rich amount of fossil evidence, as well as living taxa, reveal the remarkable morphological disparity of paired fins; such varied form is thought to be linked to the success of this broad class of vertebrates. However, fin morphology is also quite consistent even across broad taxonomic comparisons. The cause of this canalization may stem from developmental constraints in shaping morphological complexity available for selection.

It has been hypothesized that paired fins would have assisted fish with maneuvering in their aqueous environment and increased stability while in motion (Harris, 1936, 1937, 1938). Pectoral and pelvic fins in some species have even acquired novel functions, such as threatening predators, sensing taste, walking, or flying (Dasilao & Sasaki, 1998; Gosline, 1994; Harvey & Batty, 2002; Jung et al., 2018). Modifications of basal bones and distal fin rays underlie many of these changes and are tied to an individual's ability to thrive in their respective habitats. However, in the wide spectrum of fin morphologies, remarkably similar evolutionary patterns are discerned in separate lineages. Exceptionally wide paired fins, for instance, evolved in multiple teleost lineages independently (De Meyer & Geerinckx, 2014). Similar morphologies achieved in different lineages are convergent, often taking place under specific ecological demands. Despite broad recognition of these coincidences in morphology, the genetic and environmental causes underlying convergence have remained undefined. Are only specific domains of fins susceptible to change during development? Are the same genes or genetic pathways involved in convergent evolution? How does the genetic regulation of these structures bias phenotypic trends seen in evolution of fin morphologies?

Developmental constraints, which could restrict the morphospace of body patterning during the ontogeny, have been proposed as a key factor shaping the character of fin form (Beldade, Koops, & Brakefield, 2002; Cheverud, 1984; Gould & Lewontin, 1979). One of the well‐recognized causes of the constraints in development is the reuse of genes during development in new contexts. Animals repeatedly deploy the limited number of genes and genetic cascades in different developmental processes and physiological functions (Hodgkin, 1998; Williams, 1957). This pleiotropy of gene function leads to inter‐dependencies between traits and limitations on variability (Lonfat, Montavon, Darbellay, Gitto, & Duboule, 2014). One benefit of the pleiotropy is the conservation of genes and genetic pathways during evolution (He & Zhang, 2006). The repetitive use of the same genes in multiple pathways, however, imposes limitation on changes in function of those genes to maintain viability. Thus, as a by‐product of evolving a complexity, pleiotropy limits evolvability and rapid adaptation to new environments (Morris et al., 2019). Despite the common acceptance of pleiotropy as a developmental constraint, a broader understanding of the structure of developmental constraints and its implications on morphology remains incomplete due to the lack of amenable models to test hypotheses.

Rapid advancements of genomics and molecular biology make these questions within our reach, even deploying non‐model organisms into lab experiments. De novo sequencing of genomes in non‐model organisms is becoming much more frequent and attainable due to the appearance of new technologies that produce long‐read fragments (McCombie, McPherson, & Mardis, 2019). Moreover, genetic manipulations, including functional knockout and transgenesis, are expanding functional testing to non‐model organisms – fueling paradigm shifts in evolutionary developmental biology (Barman et al., 2017). With the background of these dramatic changes in experimental biology, an analysis of the underlying regulation of fin morphology, which holds both robustly conserved and extremely divergent aspects, serves as one of the prominent models to reveal underlying mechanisms of developmental constraints.

Here, we review the existing knowledge of morphology and patterns of diversity in an assessment of the evolutionary history of the paired fins, emphasizing conserved and divergent architectures. Next, we summarize the developmental processes of paired fins with underlying genetic networks, which provide fundamental insights into the developmental constraints of fins. Finally, we compare the genetic alterations responsible for extremely deviated fin morphology in teleosts and cartilaginous fishes to gain a deeper understanding of developmental constraints of fin morphology. The integration of newly emerging concepts in the evolution of paired fins sheds light on different layers of developmental constraints for fin diversity, enabling us to grasp the evolutionary trends of paired fins.

## 2 LIMITED MORPHOSPACE OF PAIRED FINS

**FIGURE 1:**
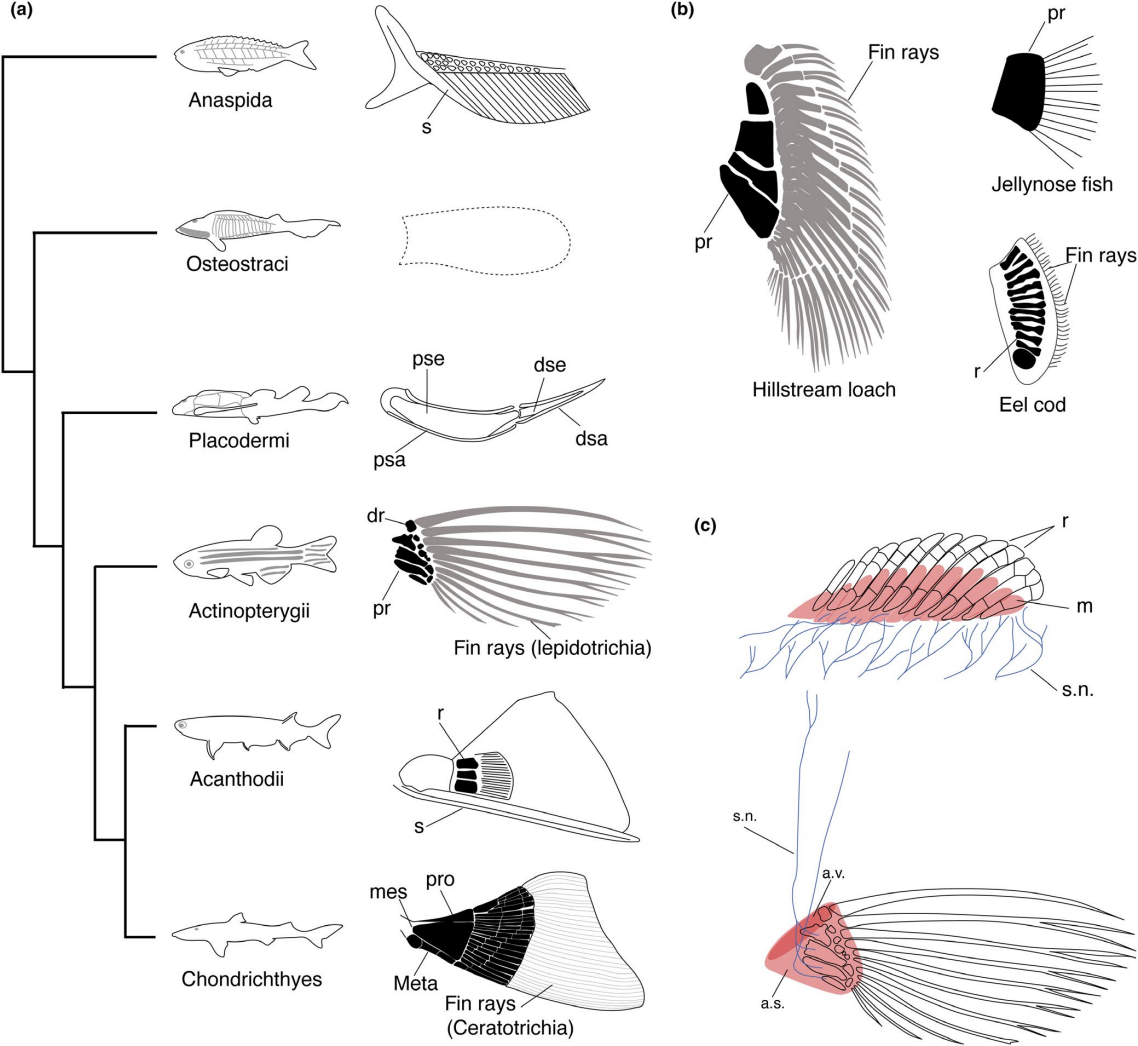
The evolutionary history of fin diversity. (a) The evolutionary trajectory of paired fins in six major fish groups. From top; *Rhyncholepis* (Anaspida), *Norselaspis* (Osteostraci), *Bothriolepis* (Placodermi), *Squalus* (Chondrichthyes), *Acanthodes* (Acanthodii), and *Danio* (Actinopterygii). *Rhyncholepis* possessed fin‐like flaps with spines (s). The length of flaps varies depending on species. Endoskeleton of *Norselaspis* fin is unknown. *Bothriolepis* evolved the pectoral fin with nerves, muscles, and blood vessels, which seem to be used for active fin movement. Later, a tribasal fin, often exhibiting fin spines, evolved in chondrichthyans and actinopterygians. (b) Diversity of paired fin skeletons. While the number of fin rays is susceptible to change during evolution, the number of proximal radials generally do not go over four in most species. The left is hillstream loach (*Beaufortia kweichowensis*) with four broad proximal radials and the right top is jelly nose fish (*Ijimaia antillarum*) with the fusion of proximal radials into one bone. The right bottom is eel cod (*Muraenolepis Kuderskii*) with 13 radials, which is not the typical number of radials in Actinopterygii. (c) Innervation and musculature of the dorsal and pectoral fin. Top; the developing dorsal fin of sharks (*Scyllium canicula*). Each muscle bud (pink) associated with a radial is innervated by a spinal nerve (blue). Bottom; the adult pectoral fin of zebrafish. Four spinal nerves innervate the fin musculature that moves fin rays. Abductor superficialis (a.s.) articulate the girdle and proximal fin rays and arrector ventralis (a.v.) connects to the first fin rays. The number of somites in paired fin development could be related to the number of proximal radials seen (see the text). a.s.; abductor superficialis, a.v.; arrector ventralis, dr; distal radial, dsa; distal segment of exoskeleton fin armor, dse; distal segment of endoskeleton, m; muscle, meso; mesopterygium, meta; metapterygium, pr; proximal radial, pro; propterygium, psa; proximal segment of exoskeleton fin armor, pse; proximal segment of endoskeleton, r; radial, and s.n.; spinal nerve. All illustrations are after: (Balushkin & Prirodina, 2007; Goodrich, 1906; Grandel & Schulte‐Merker, 1998; Hara et al., 2018; Janvier, 1996; Kardong, 2012; Matsubara, 1963; Ritchie, 1980; Schaeffer & Williams, 1977; Stensiö, 1959; Stensio, 1964; Yano et al., 2012)

It is hypothesized that the appearance of paired appendages, specifically pectoral fins, increased the body stability and optimized mobility in vertebrate evolution as fish began to explore and move around their environment (Harris, 1936). In Anaspida, a group of extinct jawless fish, some species possessed a fin‐like flap with spines posterior to the external branchial openings (Figure 1a) (Janvier, 1996). These flaps did not encompass endoskeletal components, but were constructed of fin‐ray like structures. These paired appendage structures had indications of radial muscles, although Anaspida's flaps may not represent the ancestral state of paired fins due to the possibility of their convergent evolution with paired fins of other species (Blom, 2012; Coates, 2003; Keating & Donoghue, 2016; Ritchie, 1964; Smith, 1957; Stensio, 1964) (Figure 1a). Following, Osteostraci, another group of fossil jawless vertebrates, had pectoral fins constructed of a single cartilaginous plate positioned posterolaterally to the skull shield (Janvier, 1996; Janvier, Arsenault, & Desbiens, 2004). A well‐preserved dermal skull shield of *Norselaspis*, in Osteostraci, reveals foramina for a nerve supply to the pectoral fin, suggesting the presence of muscles and nerves in the pectoral fin (Figure 1a) (Wängsjö, 1952). The endoskeletal remains of paired fins are also observed in Placodermi, a jawed fish, and most likely their fins evolved as environmental demands for robust maneuverability increased (Stensiö, 1959).

Of note, placoderm lineages, such as represented by Antiarchi, possessed simple monobasic fins, composed of one endoskeletal element, surrounded by dermal bony plates (Long, Trinajstic, & Johanson, 2009; Westoll, 1947) (Figure 1a). These paired fins appear to be innervated by spinal nerves considering the presence of foramina of the girdle bones, which indicates that placoderm groups actively move their pectoral fins with robust endoskeletons (Stensiö, 1959). Overall, Placoderms display intraspecific variation within their pectoral fins with the number of basal bones diverged from single to three in their evolution (Goujet, 2001; Goujet & Young, 2004; Westoll, 1947).

As pectoral fins continued to evolve throughout the gnathostome lineage, similar anatomical elements of the pectoral fin become shared among multiple groups (Coates, 2003). The gnathostome paired fin skeleton (excluding certain placoderms) typically consists of proximal bones and distal fin rays. The proximal bones include anteroposteriorly arrayed radials and basals, which presumably originated via the fusion of some radials (Goodrich, 1930) (Figure 1a). The fin rays are called by ceratotrichia in cartilaginous fishes (chondrichthyans, class of Chondrichthyes) or lepidotrichia in ray‐finned fishes (actinopterygians, class of Actinopterygii) (Carroll, 1988; Goodrich, 1930; Janvier, 1996) (Figure 1a). Cartilaginous fishes, and some families of ray‐finned fishes, possess three types of basal bones: the metapterygium, mesopterygium, and propterygium (arranged from the posterior to anterior), which articulate to the girdle (”Chondrichthyes” and “Acanthodii” in Figure 1a). In the teleost lineage of ray‐finned fishes, which comprises over half of all vertebrate species, it is hypothesized that they lost the ancestral metapterygium and possess only propterygium and mesopterygium (Daniel, 1934; Davis, Shubin, & Force, 2004). In teleosts, at the distal end of proximal radials, small endochondral bones, called distal radials, reside from which dermal fin rays extend off (Grandel & Schulte‐Merker, 1998) (Figure 1a). Radials and fin rays are ontogenetically and histologically different bones; radials have perichondral ossification, whereas fin rays are dermal bones that develop via intramembranous ossification without a cartilaginous stage (Hall, 2005; Wood & Nakamura, 2018).

Over time, fin structures have been remodeled by specific locomotion types; however, some features of their morphology are remarkably converged to the specific patterns. For example, in teleosts, the exceptionally wide paired fins of hillstream loach allow optimal maneuverability and overall adaptation to a fast‐moving stream environment (De Meyer & Geerinckx, 2014). Despite the peculiar size of its pectoral fin, the hillstream loach does retain a simple set of four wide proximal radials, achieving its large paired fins (De Meyer & Geerinckx, 2014) (Figure 1b). A group of gurnard also possesses large pectoral fins using them for threatening predators. Their fins stem from large, but not extra, radial elements distally (Breder, 1963; Finger & Kalil, 1985; Gosline, 1994). Generally, the number of proximal radials in teleosts is four, with some exceptions, including some eel cods or jelly nose fishes (Figure 1b) (Balushkin & Prirodina, 2007; Matsubara, 1963) while the number of fin rays often increases or decreases, even intraspecifically (Balushkin & Prirodina, 2006; Giles et al., 2015; Miller, Cloutier, & Turner, 2003) (Figure 1b). This rule does not apply to some cartilaginous fishes or Acanthodii fishes as they have more than four proximal bones (Maisey et al., 2017). For example, the pectoral fins of some sharks, skates, and rays expand anteroposteriorly and have multiple segmentations in their radials, despite the possession of the conserved tribasal patterns (Coates & Sequeira, 2001; Daniel, 1934). Thus, “the four‐basal rule” seems to be a constraint arising in stem and crown groups of teleosts.

The maximum number of the proximal radials in teleosts may depend on their developmental origins and interaction with other tissues. During teleost evolution, the size of paired fins became smaller, presumably causing overcrowding of mesenchymal cells within them, and then procartilaginous rudiments for fin radials evolutionarily fused together (Goodrich, 1930). Thus, the radials in modern teleosts develop from one continuous mesenchymal plate at the early stage and then divide into each rudiment. This derived developmental process implies that, even though modern teleost radials develop from a single large plate, the prospective procartilaginous rudiments in the plate may retain their original topological information by which they develop as completely separated bones in ancestral paired fins (Balfour, 1881; Goodrich, 1906).

Unpaired fins, such as dorsal fins, may hold the key to reveal developmental constraints on the number of proximal radials in paired appendages. Unpaired fins typically develop with a ridge of the epidermis that covers the median mesenchyme plate, which later develops the basal radials of the fin skeleton (Balfour, 1881). Importantly, each muscle bud stems from a single somite, which is innervated by spinal nerves, and is associated with one radial during unpaired fin development (Balfour, 1881; Dohrn, 1884; Mayer, 1885). The phylogenetic and comparative studies indicate that unpaired fins evolutionarily preceded paired fins in fish, which is currently explained by two debated hypotheses (Balfour, 1881; Gegenbaur, 1878; Goodrich, 1930; Mivart, 1879; Thacher, 1877). One of them, the lateral fin‐fold theory, hypothesizes that longitudinal lateral folds ultimately separated into paired appendages (Balfour, 1881; Mivart, 1879; Thacher, 1877). The morphology of hypothetical longitudinal folds is similar to that of long stretched unpaired fins, positing that the developmental programs of unpaired fins are co‐opted into the lateral folds. Therefore, the correspondence between a muscle‐bud and radial in unpaired fins may be evolutionarily maintained in paired fins (Balfour, 1877; Goodrich, 1930); following, the development of proximal radials may be shaped, or constrained, by the number of somites (Goodrich, 1906) (Figure 1c). Alternatively, the functional necessity may constrain the number of proximal radials as if excessive division of the proximal radials into smaller elements may inhibit their proper function, such as producing propulsion; there are exceptions to this generality among teleosts such as eel cods of which pectoral fins possess up to 13 proximal radials, yet these radials are embedded in a large cartilaginous plate (Balushkin & Prirodina, 2006).

## 3 GENETIC ARCHITECTURES FOR THE REGULATION OF FIN SHAPE

The fish fin is one of the prominent systems to understand developmental mechanisms of vertebrate appendages due to its thin and transparent structure (Grandel & Schulte‐Merker, 1998). While decades of studies have identified the developmental logics of tetrapod limbs mainly using mice and chickens (Mariani, Fernandez‐Teran, & Ros, 2017; Sheeba & Logan, 2017; Tickle & Towers, 2017; Zeller, López‐Ríos, & Zuniga, 2009), recent extension of gene expression studies into cartilaginous fishes and ray‐finned fishes permits interrogation of the genetic mechanisms of fin development and, through comparison, their evolution (Ahn & Ho, 2008; Dahn, Davis, Pappano, & Shubin, 2007; Davis, Dahn, & Shubin, 2007; Freitas, Gómez‐Skarmeta, & Rodrigues, 2014; Tulenko et al., 2017; Woltering, Holzem, Schneider, Nanos, & Meyer, 2018). These studies have highlighted the conserved and diverse genetic networks underlying fin evolution (Davis, 2013; Petit, Sears, & Ahituv, 2017; Zuniga, 2015). Here we highlight a few mechanisms and variation in their use through development of diverse fins.

### 3.1 Early fin initiation

**FIGURE 2:**
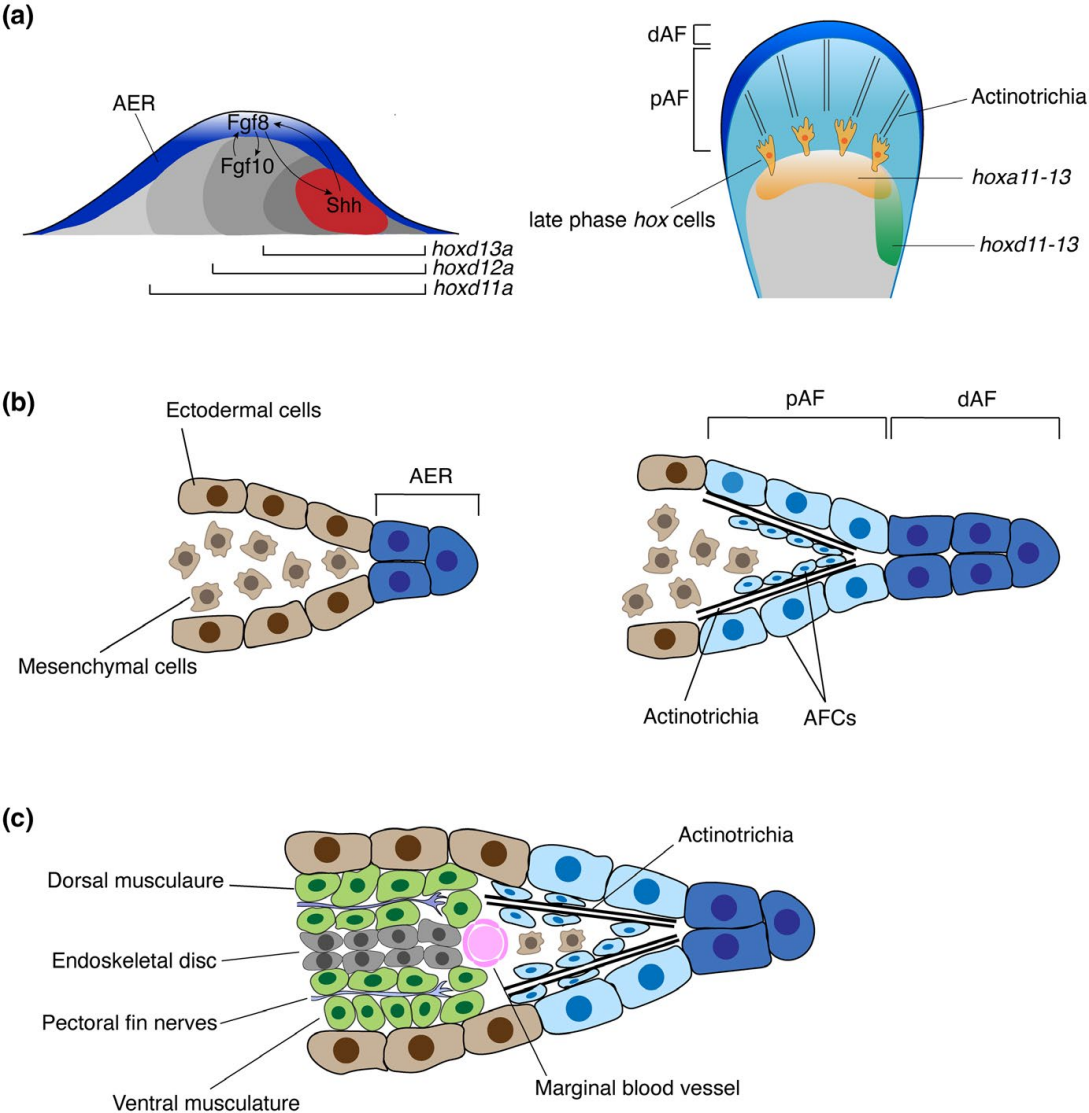
The developmental mechanisms of fish fins. (a) Developmental mechanisms of the pectoral fin. Left; at the early stage, apical ectodermal ridge (AER) expresses Fgf8 and other growth factors which stimulate cell proliferation via the positive feedback with Shh in ZPA. *Hox* genes establish the nested expression patterns from the posterior to anterior fin and set positional information for fin patterning. Right; at the late stage, AER transforms into apical fold (AF), in which actinotrichia develop. *Hoxa13* genes are expressed in the distal mesenchyme of the endoskeletal disc and these cell populations migrate into the AF. *Hoxd11‐d13* genes are expressed in the posterior mesenchyme and regulate skeletal patterning (Freitas, Gómez‐Marín, Wilson, Casares, & Gómez‐Skarmeta, 2012; Nakamura et al., 2016). (b) The diagram for the transformation of AER into AF in unpaired fins. AER establishes the thickened ectodermal layer during the early fin development. Then, AER changes to AF, in which the distal Apical Fold (dAF) forms two layers of the ectodermal tissue. Once AER transforms into AF, mesenchymal cells migrate into the proximal Apical Fold (pAF). Actinotrichia Forming Cells (AFC) which express *And1* gene are in the ectoderm and mesenchyme, developing actinotrichia. (c) Concurrent development of skeletons, muscles, and nerves around 120 hpf of zebrafish pectoral fin. In the proximal fin, the endoskeletal disc consists of two layers of cells. Muscle cells are at the dorsal and ventral to the endoskeletal disc, and will give rise to adductor and abductor muscles later. Marginal blood vessel is at the distal to the endoskeletal disc. Spinal nerves enter the pectoral fin and innervate dorsal and ventral fin muscles (Grandel & Schulte‐Merker, 1998)

The growth of the fin bud occurs in the lateral plate mesoderm (LPM). *Tbx5,* a T‐box transcription factor, of which mutations cause heart and limb defects such as Holt‐Oram syndrome (Bruneau et al., 2001), is one of the early markers of pectoral fin formation (Figure 2a). Previous studies showed that the expression pattern and function of Tbx5 in the pectoral fin/forelimb domain are conserved from fish to tetrapods as an ancient feature of jawed vertebrates (Adachi, Robinson, Goolsbee, & Shubin, 2016; Ahn, Kourakis, Rohde, Slivert, & Ho, 2002; Pi‐Roig, Martin‐Blanco, & Minguillon, 2014; Tamura, Yonei‐Tamura, & Belmonte, 1999). Without Tbx5 function, the fin bud development fails and all its derivatives, including the pectoral girdle, are malformed (Ahn et al., 2002; Garrity, Childs, & Fishman, 2002; Mao, Stinnett, & Ho, 2015).

Once the fin starts to grow, the Apical Ectodermal Ridge (AER), a thickened ectodermal structure, is formed along the distal edge of the fin bud overlying the mesenchymal cells (Grandel & Schulte‐Merker, 1998) (Figure 2a). The fin AER stimulates bud outgrowth in the distal direction, expressing key growth factors such as *Fibroblast growth factor 8* or *Wnt 3*, which promote the permissive growth of the fin bud. Disruption of these signaling functions in AER formation results in the early truncation of the fin (Fischer, Draper, & Neumann, 2003; Nagayoshi et al., 2008). Growth and positional information along the anteroposterior (AP) axis in fin/limb development are accurately coordinated by another signaling center which is located in the posterior fin mesenchyme, Zone of Polarizing Activity (ZPA) (Akimenko & Ekker, 1995; Hoffman, Miles, Avaron, Laforest, & Akimenko, 2002) (Figure 2a). In ZPA, Sonic Hedgehog (Shh), a secreted signaling protein, is expressed and sets a gradient of the signaling across the fin bud, providing the AP positional information with stimulating fin growth (Ahn & Joyner, 2004; Akimenko & Ekker, 1995; Harfe et al., 2004). The effect of Shh on fin patterning is evolutionarily conserved with tetrapod limb; functional perturbation or knockout analysis of *Shh* in chondrichthyans and teleost fishes shows the loss of AP patterning and reduction of the fin size (Dahn et al., 2007; Neumann, Grandel, Gaffield, Schulte‐Merker, & Nüsslein‐Volhard, 1999), whereas its upregulation exerts opposite effects (Chen, Wang, Yu, Wu, & Pai, 2009). Though the expression of *Shh* in the posterior fin is conserved among bony and cartilaginous fishes, the timing of onset varies, contributing to the fin/limb shape diversity (Dahn et al., 2007; Sakamoto et al., 2009; Shapiro, Hanken, & Rosenthal, 2003).

The Shh activity from ZPA is also required for the proximodistal fin growth in fishes by establishing the positive feedback loop with AER (Niswander, Jeffrey, Martin, & Tickle, 1994; Zeller et al., 2009). Shh expressed in ZPA induces Fgf8 expression in AER and*,* in turn, Fgf8 upregulates Shh, creating a reciprocal signaling loop critical for the expansion of the fin bud (Figure 2a) (Mercader, 2007; Nomura et al., 2006). The loss of components in this feedback loop leads to severe phenotypes in fin development; *Fgf10* mutant zebrafish (*Danio rerio*) have defective AER formation, resulting in a truncated pectoral fin (Norton, Ledin, Grandel, & Neumann, 2005). This feedback loop between AER and ZPA is most likely conserved in other fishes, as skate pectoral fin displays similar expression patterns of *Fgf8* and *Fgf10* in AER and underlying mesenchyme, respectively (Nakamura et al., 2015). As the alteration of *Shh* or *Ptch1* expression patterns leads to changes of digit number in tetrapods (Lopez‐Rios et al., 2014), fine tunings of the parameters in this conserved feedback loop are likely to underlie the diversity of paired fin shape.

### 3.2 Extension of fin growth and patterning

In contrast to tetrapod limbs that develop exclusively with AER, fins of chondrichthyans and actinopterygians transform the early AER to an extended ectodermal structure called the Apical Fold (AF) (Figure 2a,b) (Thorogood, 1991; Yano, Abe, Yokoyama, Kawakami, & Tamura, 2012). The thickened ectodermal structure of the AER separates into two layers as the distal fin mesenchyme distally migrates between these two layers, extending the AF domain (Figure 2b) (Yano et al., 2012). Subsequently, actinotrichia forming cells (AFC) differentiate, of which the developmental origin remains elusive, and form the actinotrichia, the embryonic predecessor of the fin rays (Figure 2b) (Durán, Marí‐Beffa, Santamaría, Becerra, & Santos‐Ruiz, 2011). Actinotrichia is an assembly of collagen and non‐collagen components synthesized by *and* gene products (Zhang et al., 2010). During later development, actinotrichia is replaced by the dermal bones called lepidotrichia, a major component of fin rays.

Recent genetic studies have highlighted remarkable conservation and functional differences of key developmental genes in appendage diversity, including the tetrapod distal limb (prospective endochondral bones) and fish AF (prospective dermal fin rays), which are ontogenetically and structurally different. *Hox* transcription factors are central to body patterning in vertebrates (Deschamps & van Nes, 2005; Mallo, 2018; Young & Deschamps, 2009) and exhibit nested expression patterns in appendage primordia (Figure 2a) (Pérez‐Gómez, Haro, Fernández‐Guerrero, Bastida, & Ros, 2018; Zakany & Duboule, 2007). Strikingly, comparative studies in catshark (Freitas, Zhang, & Cohn, 2007), paddlefish (Davis et al., 2007; Tulenko et al., 2016), medaka (Takamatsu et al., 2007), and zebrafish (Ahn & Ho, 2008) all showed nested expression patterns of *Hoxa* and *d* genes in the endochondral disc and even in the AF, emphasizing that nested *Hox* expression patterns in appendage development are deeply conserved features from fins to limbs beyond their apparent morphological disparity (Davis, 2013; Lalonde & Akimenko, 2018; Tulenko et al., 2016). Not only *Hox* genes but also other gene expressions such as *Ectodysplasin receptor (Edar)* are conserved between fish fins and mouse limbs. *Edar* is expressed in the distal endochondral radials and forming lepidotrichial rays, and the mutation in *Edar* gene disrupts the development of radials and fin rays in zebrafish (Harris et al., 2008). In the mouse limb bud, it is expressed in AER and necessary for the sweat gland formation (Pispa, Mikkola, Mustonen, & Thesleff, 2003). Given that the mouse limb only consists of endochondral bones without fin rays, the downstream networks of *Edar* in lepidotrichial rays seems to be lost from appendages during the fin‐to‐limb transition. These unexpected conservations of tool‐kit genes in the AF and tetrapod limbs imply that the ground plan of the AF domain is established via the conserved genetic mechanisms among diverse appendages. Yet modifications of their downstream network could have produced morphological diversity such as fins and limbs (Nakamura, Gehrke, Lemberg, Szymaszek, & Shubin, 2016).

In contrast to the conserved repertoire of genes active in appendage development and their evolution, fin development also deploys lineage‐specific mechanisms. In teleosts, diversification of Fgf signaling underlies early divergence. The fish‐specific gene *Fgf24* (Jovelin et al., 2010), which is expressed in the early fin bud mesenchyme and late fin AER, activates *Fgf10* expression and regulates cell migrations into the fin bud (Fischer et al., 2003; Mao et al., 2015). The *Fgf24* mutant fish (*ikaraus*) results in the complete absence of the pectoral fin. Intriguingly, *Fgf24* was lost during the fish‐to‐tetrapod transition and could be critical for the fin‐to‐limb change. Moreover, zebrafish *Fgf16* is indispensable for the proliferation of mesenchymal cells and differentiation of AER, but its function for the tetrapod limb development is unknown (Nomura et al., 2006). These genetic mechanisms for fin development could be driving factors of fin diversity in teleost fishes, and even in other fish including cartilaginous fishes.

### 3.3 In patterning the fin

In fin development, skeletons, muscles, and nerves form under the precise coordination in a spatial and temporal manner (Figure 2c) (Thorsen & Hale, 2005; Thorsen & Hale, 2007). The pectoral fin of zebrafish develops in the LPM adjacent to the somite one to four (Mao et al., 2015) and other teleosts develop their pectoral fins at the comparable position, at least at the initial stage of fin development (Ahn et al., 2002; Richards, 2005). Muscle precursor cells develop from the somitic mesoderm, and in zebrafish, cells from somites two to four migrate into the pectoral fin bud with the expression of *Lbx2*, a homologue of amniote *Lbx1* and a marker of migrating muscle precursor cells (Neyt et al., 2000; Ochi & Westerfield, 2009) (Figure 2c). Muscle development through the migration of *Lbx2*‐positive cells occurs as skeletal development with the mesenchyme of the LPM and blood vessel development proceeds (Grandel & Schulte‐Merker, 1998). This suggests that the fin bud grows as a highly heterogeneous tissue with physical and molecular interactions among multiple types of cells (Figure 2c).

Intriguingly, in tetrapods, the paraxial mesoderm cells and LPM cells retain their original topological relationship throughout the development of the girdle which supports the limbs at their proximal end (Huang, Zhi, Patel, Wilting, & Christ, 2000; Shearman, Tulenko, & Burke, 2011; Wang et al., 2005). An analogous process occurs during the development of pectoral fins in cartilaginous fish, where muscles and nerves develop closely associated with forming rays (Goodrich, 1930; Lopez‐Rios et al., 2014; Turner et al., 2019), maintaining their segmental order. Given that the somitic mesoderm and LPM in adjacent positions interact by diffusible molecules such as retinoic acid and Wnts (Gibert, Gajewski, Meyer, & Begemann, 2006) which, in turn, affect collinear *Hox* expression patterns (Prince, Joly, Ekker, & Ho, 1998), it would be intriguing to test whether the skeletons originating from the LPM are affected by signaling from the somitic mesoderm or by physical interactions between these tissues. Testing the mutual effects between LPM and the somatic mesoderm during fin development would help to answer how skeletons and muscles simultaneously evolve to achieve functional fin structures in the evolution of morphological diversity in paired fins.

## 4 RELEASE FROM DEVELOPMENTAL CONSTRAINTS – FINS OF BENTHIC DWELLERS

Adaption to benthic habitats is one of the fascinating examples of fin evolution, in which the size of the pectoral fins repeatedly and independently expands in multiple lineages (Cooper et al., 2010; Muschick, Indermaur, & Salzburger, 2012; Recknagel, Elmer, & Meyer, 2014). Wide fins exert novel essential functions for fish survival, such as burying body into sand (Hauser, 2011), threatening predators (Gosline, 1994), or clinging rocks in fast currents (De Meyer & Geerinckx, 2014). Recent careful examinations of their anatomy and development highlight distinct evolutionary modes and genetic differences between teleosts and cartilaginous fishes, shedding light on developmental potential and constraints in fins.

**FIGURE 3:**
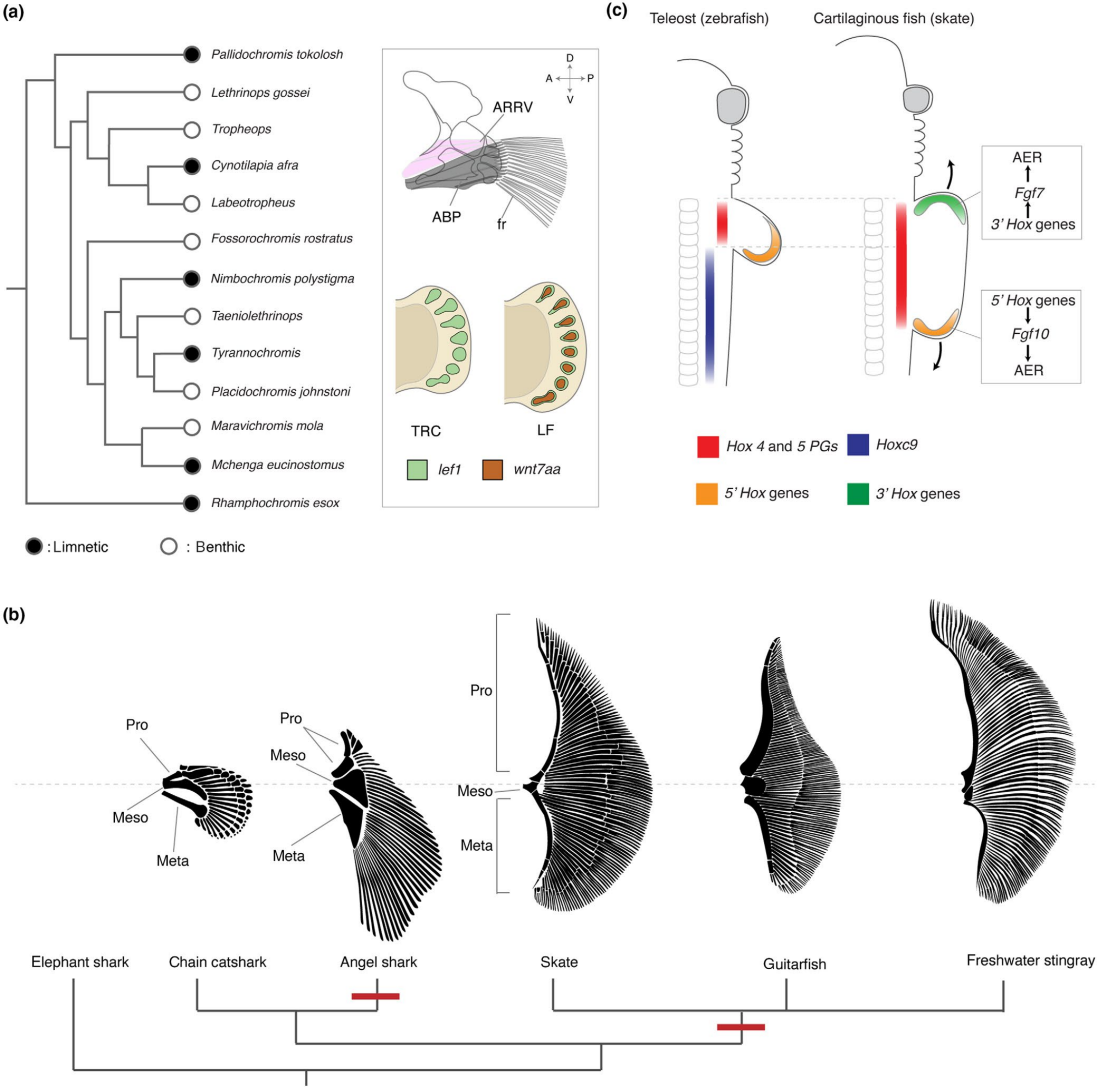
Distinct mechanisms of the fin expansion in teleosts and cartilaginous fishes. (a) The recurrent evolution of wide paired fins in African cichlid fishes. Black circles indicate limnetic species and white circles indicate benthic species that evolved in separate lineages in multiple times (Hulsey et al., 2013). Right box; the generalized adult pectoral fin of cichlid fish (Hulsey et al., 2013) and the expression pattern of *Lef1* and *Wnt7aa* in the developing pectoral fins of LF and TRC (Navon et al., 2017). The dorsoventral and anteroposterior axes are indicated. (b) Repeated evolution of wide paired fins in cartilaginous fishes. Note that basal bones (propterygium, mesopterygium, and metapterygium) evolved to be very wide in angelsharks, skates, guitarfishes, and stingrays. Wide paired fins of angelsharks and rays are the consequence of convergent evolution (red nodes). (c) Developmental mechanisms of paired fins in zebrafish (teleosts) and skates (cartilaginous fish). In teleosts, *Hox4* and *5* PGs (red) are expressed in the LPM and are likely important to induce the fin bud as tetrapods. *Hoxc9* (blue), which functions as a repressor of *Tbx5* induction in tetrapods, is expressed in the posterior lateral plate mesoderm. However, in cartilaginous fish, *HoxC* cluster genes were decreased or completely lost. In addition, skate embryos exhibit the reorganization of *Hox* expression patterns, such as the expansion of *Hox4* and *5* to the posterior body, evolving strikingly wide fins. In the pectoral fin, skates possess 3′*Hox* module which anteriorly extends the fin, in addition to 5′*Hox* module which elongates the fin to the posterior direction. ABS; abductor superficialis, AR; arrector ventralis, and fr; fin rays, meso; mesopterygium, meta; metapterygium, and pro; propterygium. All illustrations are after: (Comer, Klochko, Pauly, Cousteau, & Parenti, 2008; Carvalho et al., 2008; Ebert & Gon, 2017; Hulsey et al., 2013; Nakamura et al., 2015)

Several species of cichlids, living in African lakes, have independently adapted to benthic habitats for foraging and show convergent morphological trends; the number of fin rays is increased and they have a concordant widening of the abductor superficialis (ABS), the muscle which articulates the girdle to the proximal fin rays (Figure 3a) (Hulsey, Roberts, Loh, Rupp, & Streelman, 2013). To reveal genetic basis underlying this recurrent evolution of benthic fin morphology, Quantitative Trait Loci (QTL) was employed (Navon, Olearczyk, & Albertson, 2017). Two benthic species, blue mbuna (*Labeotropheus fuelleborni*:LF) and *Tropheops* sp. ‘red cheek’ (TRC), in which the pectoral fin in LF possesses more fin rays than that in TRC, were crossed with each other and their F2 were analyzed by morphometrics with mapping to identify responsible loci for the ‘wide fin’ phenotypes (Navon et al., 2017). One of the identified single nucleotide polymorphisms (SNPs) is located approximately 40 kbp away from *Wnt7aa*, of which homologue is expressed in the dorsal ectoderm of the tetrapod limb regulating the dorsoventral asymmetry (Kengaku et al., 1998; Parr, Shea, Vassileva, & McMahon, 1993). Because *Wnt7aa* expression was found in the developing fin of LF but not in that of TRC*,* the gain of novel *Wnt7aa* expression domain seems to evolve the wide fin in LF (Figure 3a). Moreover, alterations of several other gene expressions as well as *Wnt7aa* might cooperatively contribute to the wide paired fins in cichlids as a SNP close to *Col1a1*(type 1 collagen gene), a bone differentiation marker (Fisher, Jagadeeswaran, & Halpern, 2003), was also identified as a loci associating with wide fin phenotypes. This raises a possibility that the alteration of expression levels of multiple genes might synergistically produce the evolutionary diversity of the fin width. The next challenge would be to test how *Wnt7aa*, *Col1a1*, and other genes have been involved in convergent evolution of benthic teleost fins, such as hillstream loach; whether all the same genes, some of them, or utterly different gene sets are involved in convergent evolution of wide fins.

Cartilaginous fish, including skate and rays (Batoidea), and angelsharks (Selachii), also evolved wide paired fins for benthic habitats (Figure 3b), however, their developmental and genetic basis seems to be fundamentally different from ones found in teleosts. Exceptionally wide paired fins of skates develop from the significantly wide fin bud and constitute a large part of the flat body (Martinez, Rohlf, & Frisk, 2016). Despite their exceedingly deviated shape from other fins, the internal skeletons primarily consist of three basal bones: metapterygium, mesopterygium, and propterygium (Figure 1a, Figure 3b). Intriguingly, skates and rays, and angelsharks independently achieved their peculiar but similar wide fins with comparable skeletal architectures from their common ancestors (Carrier, Musick, & Heithaus, 2012) – a striking example of convergent evolution.

In the LPM of tetrapods, Hox4 and Hox5 paralogous groups (PGs) were suggested to induce *Tbx5* expression (Minguillon et al., 2012), which promotes the limb growth (Agarwal et al., 2003; Ahn et al., 2002; Rallis et al., 2003). To restrict the expression domain of *Tbx5* to a certain degree in the LPM, Hoxc9 plays an opposite role, repressing *Tbx5* expression via the competitive binding to the *Tbx5* enhancer (Nishimoto, Minguillon, Wood, & Logan, 2014). Notably, in skates, the expression patterns of *Hox* family genes, including *Hox4*, *5*, and 9 PGs, have been extensively reorganized (Figure 3c). In situ hybridization and immunofluorescence staining identified that the expression domains of *Hox4* and *5* PGs are wider in skate embryos than those of amniotes (Jung et al., 2018; Turner et al., 2019), suggesting that the evolution of wide pectoral fins in skates attributes to the expansion of *Hox4* and *5* PGs domains. Furthermore, posterior *Hox* genes (5' *Hox* genes, such as *Hoxa9*) shifted to far more posterior, which is also likely to contribute to the fin expansion synergistically. Intriguingly, the upstream regulators of *Hox* genes in skates, such as *Raldh2*, *Wnt3*, and *Fgf8*, exhibited comparable expression patterns with those of other vertebrates, implying that *cis*‐regulatory changes of *Hox* genes underlie the posterior shifts of the nested expression patterns in cartilaginous fish (Turner et al., 2019).

During late development of the fin, the pectoral fin bud of skates further expands along the AP axis from the wide fin bud. The molecular mechanisms for this late expansion of the pectoral fin have been uncovered as well as the genetic mechanisms underlying generation of a wide fin bud. (Barry & Crow, 2017; Nakamura et al., 2015). RNA‐sequencing and subsequent in situ hybridization revealed that skate pectoral fin possesses an extra AER in the anterior fin in addition to the canonical AER that limbed vertebrates form. The posterior AER in skate fins is regulated by the conserved 5' *Hox* module, in which 5′ *Hox* genes induce *Fgf10* expression and then *Fgf10* upregulates the expression of AER genes such as *Fgf8* or *Wnt3* (Sheth et al., 2013). In contrast, the anterior AER in skate is maintained by 3' *Hox* genetic network in which 3′*Hox* genes regulate the expression of *Fgf7*. Intriguingly, Fgf7 binds to the same Fgf receptor as Fgf10, which is a component of the canonical 5′*Hox* module (Jin, Wu, Bellusci, & Zhang, 2019; Nakamura et al., 2015; Sheth et al., 2013) (Figure 3c). Thus, despite the differences of involved genes in 5′*Hox* and 3′ *Hox* genetic networks, two distinct modules achieve morphologically similar structures in the anterior and posterior fin formation.

The convergent evolution of wide paired fins in teleosts and cartilaginous fishes highlights possible developmental constraints on fin evolvability. Whereas repeated evolution of wide paired fins in teleosts mainly has modified the length and number of fin rays without drastic changes of the number of proximal radials (De Meyer & Geerinckx, 2014; Klingenberg & Ekau, 1996), cartilaginous fishes have evolved wide paired fins with significant modifications of the number of basal bones. Despite the limited information on genetic mechanisms of fin development, this difference may originate from the erosion or loss of *HoxC* cluster genes in sharks and rays (Hara et al., 2018; King, Gillis, Carlisle, & Dahn, 2011). As *HoxC* cluster genes are suggested to repress *Tbx5* expression in the LPM during limb development (Nishimoto et al., 2014), the decrease or complete loss of *HoxC* genes in cartilaginous fish provides fins opportunities to escape from the ontogenetic restriction of fin base width, increasing morphospace of pectoral fins. However, as knockout mice of the entire *HoxC* cluster did not show obvious phenotypes in appendage width but with homeotic shifts of vertebrae (Saegusa, Takahashi, Noguchi, & Suemori, 1996; Suemori & Noguchi, 2000), not only the release from HoxC repression but also the expansion of *Hox4* or *5* domains seems to be imperative for evolution of wide fins. Accordingly, HoxC repression is hypothesized to be one of the developmental constraints that limit the evolvability of fin width and the loss of HoxC genes might grant an evolutionary possibility for wide fins to cartilaginous fish such as by expanding *Hox4* and *5* PG domains.

## 5 TOWARDS A DEVELOPMENTAL EVOLUTIONARY MODEL OF FIN DIVERSITY

Cumulative knowledge from disparate disciplines highlights distinct aspects of developmental constraints that shape fin evolution operating at different levels of development such as tissue and cell behavior. Only through the integration of anatomy, embryology, and comparative genomics are we able to understand evolutionary trends underlying transitions in fin diversity. Here, we summarize current hypotheses and next questions for the developmental constraints of fin evolution.

### 5.1 The primary shape of paired fins is predictable by the width of fin bud base

The fin shape of cartilaginous fishes is extraordinarily diverse as skates and rays exhibit the most extreme pectoral fin forms of the group (Carrier et al., 2012; Daniel, 1934). This diversity is produced during the ontogenetic process; the width of early fin bud is likely determined by Hox genes, and then the fin anteroposteriorly extends during later development (Maxwell, Fröbisch, & Heppleston, 2008; Nakamura et al., 2015; Turner et al., 2019). Intriguingly, the width of the fin anlagen and the AP elongation of the fin, seem to be positively correlated; the width of fin attachment site to the body trunk in angelshark pectoral fin is slightly wider and the fin extends more anteriorly than other sharks, whereas skates and stingrays show the extraordinarily wide fin bud at the early developmental stage with the full elongation of the fin to the head at the late stage (Carvalho, Kriwet, & Thies, 2008; Maxwell et al., 2008). Although the genetic linkages of these two different developmental processes are unknown, it appears to be possible to predict somewhat how long the pectoral fins extend towards the head by observing the width of fin anlagen. The same genes or genetic pathways may be involved in both cases; hox genes that regulate the width of fin bud anlagen, for example, could also be involved in the anterior elongation of the fin.

### 5.2 The number of radials is regulated by species‐specific genetic modifications

Cartilaginous fish, including extinct sharks, exhibit remarkable diversity in the number of basal bones. Some extinct species, such as *Antarctilamna* or *Expleuracanthus,* possessed a long series of basal radial bones within the pectoral fin – the segmented metapterygium (Janvier, 1996). The number of basal bones is partly correlated to the overall size and shape of the fin as it increases with the fin size. However, given the specific alterations of branching and segmentation patterns of fin cartilages by retinoic acid treatment (Dahn et al., 2007), each bone morphology must be specified in a dynamic fashion that occurs in a taxon‐specific manner. This fact underscores the necessity for thorough comprehensive and comparative studies to fully resolve the underlying mechanisms. In contrast to cartilaginous fish, the number of proximal radials in modern teleosts is conserved up to four. The upper limit of the radial number may be an attribute to the number of somites that contribute to the fin development. This process of constraining the radial number and decoupling the act of increasing radial number and fin size in teleost fishes hinders simple prediction of fin shape by radial number (Balushkin & Prirodina, 2006). Continuous efforts, including fine mapping of genetic loci responsible for fin diversity, will provide a comprehensive view of the genetic regulation that generates radial variations in teleosts (Kawajiri, Fujimoto, Yoshida, Yamahira, & Kitano, 2015; Keong, Siraj, Daud, Panandam, & Rahman, 2014; Navon et al., 2017).

### 5.3 The correlated evolution of associated tissues in fins

For any novel morphology to function, the complex interactions of multiple tissues need to be coordinated to permit functional utility. How such coordination occurs in development is a conundrum ‐ how do different tissues such as skeletons, muscles and nerves simultaneously evolve and how do interactions among these structures constrain their transformation with each other during evolution? (Tsutsumi, Tran, & Cooper, 2017). Recent progress showed that the proximodistal pattern of muscles, joints, and skeletons are all integrated through common genetic signaling pathways in part regulated by *Hox11* genes, suggesting that these integrated tissue interactions may be in part an emergent property of development (Hawkins, Henke, & Harris, 2019). An understanding of how such integration is regulated will be insightful and add additional layers to our understanding of developmental constraints on fin morphology.

### 5.4 Fin diversity hidden by selection

The ultimate understanding of fin diversity with the underlying developmental constraints may originate from an assimilation of evolutionary biology, ecology, and embryology. Since fin morphology has been a key character under selective pressures in many differing environments, such as water, land, and air, for greater than 500 million years, the ecological niche consistently biases evolutionary trajectories of fins, thus shaping specific underlying developmental programs. The concept of "developmental constraints" can be used to help understand the types of form and diversity seen in evolution. Yet, if we can release this constraint, as can be done in a laboratory setting, we would endeavor to decipher how much evolutionary possibility is invisible to us by natural selections. A direct comparison to natural forms can reveal the broader spectrum of what development can do, the constraints development imposes on form, and ecological constrictions on developmental potential.

## CONFLICT OF INTEREST

The authors declare that this review was written in the absence of any commercial or financial relationships that could be construed as a potential conflict of interest.

## AUTHOR CONTRIBUTIONS

AE, KF, SM, NT, and TN wrote the manuscript and made figures with illustrations.
